# Completion Pneumonectomy for Non-Small-Cell Lung Cancer: Does Induction Treatment Influence Postoperative Outcomes?

**DOI:** 10.3390/cancers14143408

**Published:** 2022-07-13

**Authors:** Domenico Galetta, Lorenzo Spaggiari

**Affiliations:** 1 Division of Thoracic Surgery, European Institute of Oncology IRCCS, 20141 Milan, Italy; lorenzo.spaggiari@ieo.it; 2 Department of Oncology and Hematology-Oncology-DIPO, University of Milan, 20122 Milan, Italy

**Keywords:** lung cancer surgery, pneumonectomy, chemotherapy

## Abstract

**Simple Summary:**

In recent years there have been important improvements in surgical and adjuvant therapy for lung cancer which have led to an increasing number of patients with non-small-cell lung cancer (NSCLC) which had been previously cured by surgery being identified as having a second primary NSCLC or a recurrence of the previous tumor. In these cases, a completion pneumonectomy (CP), defined as the complete removal of the remaining lung after an ipsilateral pulmonary resection, may be performed. Although this procedure has a higher morbidity and mortality than standard pneumonectomy due to the high degree of surgical difficulty strongly associated with the previous surgery, the number of patients undergoing CP is increasing with improvement in morbidity and mortality. To the best of our knowledge, there is no study evaluating the role of induction therapy (IT) on the outcomes of patients who have undergone CP. We reviewed our single-center experience in patients receiving CP for recurrent/second NSCLC after IT and analyzed perioperative results and long-term outcomes. Our results revealed that postoperative complications were not influenced by IT, and long-term survival was adversely influenced by the absence of IT, the presence of squamous cell carcinoma, and cancers at advanced stages. Correct patient selection is crucial to evaluating possible contraindications and adopting technical details to reduce the complication rate.

**Abstract:**

Background: Completion pneumonectomy (CP) is associated with high morbidity and mortality. We reviewed our experience to evaluate whether induction treatment (IT) may affect postoperative outcomes and analyzed factors influencing long-term results. Methods: Between 1998 and 2020, 69 patients with lung cancer underwent CP (50 males, median age 63 years, right CP in 47 patients). A total of 23 patients (33.3%) received IT (chemotherapy in 15, chemoradiotherapy in 7, and radiation in 1). Surgery included 25 (36.2%) extended resections and five (7.2%) tracheal sleeve CP. Results: The 30-day mortality rate was 7.2% (5/69), and overall morbidity was 37.6%. Major complications occurred in five patients (7.2%): one cardiac dislocation, one diaphragmatic hernia, one transient ischemic attack (TIA), and two bronchopleural fistulas. Minor complications occurred in 21 cases (30.4%): pulmonary in 12, cardiac in 7, and neurological in 2. The median hospital stay was 8 days (range, 5–56 days). IT did not influence postoperative morbidity and mortality. Pathological staging included 19 (27.5%) stage I, 36 (52.2%) stage II, and 14 (20.3%) stage III. Overall 5-year survival was 51.7%. Factors influencing survival were IT (*p* = 0.01), extension of resection (*p* = 0.04), histology (*p* = 0.01), pathological stage (*p* = 0.03), and T and N factors (*p* = 0.2, respectively). Factors affecting survival in multivariate analysis included IT (*p* = 0.02) and histology (*p* = 0.03). Conclusions: In our experience, CP had a low mortality, acceptable morbidity, and good long-term survival, which justifies this surgical procedure. Postoperative complications were not influenced by IT. Long-term survival was adversely influenced by the absence of IT, the presence of extended resection, the presence of squamous cell carcinoma, and cancers at advanced stages.

## 1. Introduction

Tumor recurrence or a second tumor after a previous resection for non-small-cell lung cancer (NSCLC) may occur in 30–70% of patients [1]. It represents one of the most important factors affecting survival after NSCLC resection, which still remains less than 50% [1]. In these cases, a completion pneumonectomy (CP), defined as the complete removal of the remaining lung after an ipsilateral pulmonary resection (usually after lobectomy or bilobectomy) [2,3], may be performed [1,3,4,5]. This procedure has a higher morbidity and mortality than standard pneumonectomy [3] due to the high degree of surgical difficulty strongly associated with the previous surgery (i.e., presence of dense adhesions or fibrosis in the hilum structures). Published studies demonstrate an overall complication rate for CP performed for NSCLC ranging from 3.8% to 46.9% and operative mortality ranging from 0% to 17.6% [6]. Nevertheless, due to the rising incidence of lung cancer and thanks to the extended survival time after resection, the number of patients undergoing CP is increasing; moreover, the improvement in recent years of preoperative workup tests, anesthesia techniques, and postoperative management have led to a reduction in CP-related morbidity and mortality rates.

In the English-language literature there are few publications dealing with survival and the risk factors linked to morbidity and mortality following CP, which remains a high-risk surgical procedure. For this reason, CP is recommended only for selected patients and performed by experienced surgeons at high-volume and experienced centers [7,8].

To the best of our knowledge, there is no study evaluating the role of induction therapy (IT) on the outcomes of patients who have undergone CP. We sought to review our single-center experience in patients receiving CP for recurrent/second NSCLC after IT, analyzing perioperative results and long-term outcomes.

## 2. Materials and Methods

The study was conducted according to the guidelines of the Declaration of Helsinki; The Ethics Committee of our Institution waived the need for ethics approval and the need to obtain consent for the collection, analysis, and publication of the retrospectively obtained and anonymized data for this non-interventional study. Written informed consent was obtained from all patients. This study was reported based on the Strengthening the Reporting of Observational Studies in Epidemiology (STROBE) checklist for cross-sectional studies [9].

We retrospectively reviewed clinical records of 69 consecutive patients who underwent CP for NSCLC between January 1998 and December 2020 in our division.

CP is defined as a surgical intervention where the remaining lung parenchyma is removed after a prior ipsilateral anatomic resection (segmentectomy, lobectomy, or bilobectomy). Patients who had received a prior wedge resection were not considered in this study.

The total number of patients who underwent CP was then further subdivided into two groups: the first group received induction therapy before resection (IT group), whereas the second group underwent CP without preoperative therapy (no-IT group).

Our prospectively maintained database was used to obtain information about the following patient demographics: age, sex, respiratory function (forced vital capacity, forced expiratory volume in 1 s, carbon monoxide lung diffusion capacity), histology, type of resection, pathological stages, co-morbidities, mortality, complications, intensive care unit stay, hospital stay, and survival. Follow--up was obtained from clinical notes.

### 2.1. Clinical Considerations

All patients who underwent CP were initially clinically evaluated: patient histories were registered, and performance status and physical examination were noted. The diagnostic was performed and included the confirmation of diagnosis by fine-needle aspiration biopsy, endobronchial biopsy, or bronchoscopic brushing. The staging workup included computed tomography (CT) of the chest, abdomen, and brain as well as positron emission tomography (PET) scan. Mediastinal nodal involvement was assessed in cases of suspected of N2 disease on CT scan or PET scan by cervical mediastinoscopy or endobronchial ultra sound (EBUS).

Distinction between a second primary lung cancer and local recurrence was made according to the Martini and Melamed criteria [10]: a *local recurrence* was defined as a second malignant tumor with the same cell type occurring in the same anatomic site within 2 years of the first operation, and a *second primary lung cancer* was defined as a second malignant tumor when the cell type was different or when a tumor with same cell type occurred in a different anatomic site more than 2 years after the first cancer in the absence of residual tumor after the first operation.

IT was administered to all patients who were candidates to receive CP with a potentially resectable T4 disease (tumor invading the carina or the tracheo-bronchial angle or left atrium or aorta) with or without N2 disease. In our practice, patients with nodal involvement of mediastinal lymph node station #2R or #2L or with mediastinal lymph node station #4R bulky disease are considered unsuitable for surgery, while patients with mediastinal lymph node station #4R (no bulky) adenopathy are considered suitable for surgical resection after IT.

The no-IT group included patients without clinical mediastinal nodal involvement, those with clinical T3 (chest wall), and staged IIB or IIIA (T3 N0 or N1, respectively) (8th TNM staging system) [11].

The IT group received a platinum-based chemotherapy (cisplatin/gemcitabine or vinorelbine or taxol) or chemoradiotherapy. One patient received preoperative radiotherapy. Radiotherapy was delivered to a dose of 1.8 Gy per day over a 4 or 5-week period (total radiation dose of 50.4 Gy). All patients receiving IT were re-staged to assess clinical response and to rule out interval progression of disease before preoperative intervention. Patients repeated cardiologic evaluation, function tests, whole body CT scan, and PET scan.

The group of patients receiving IT and operated on underwent thoracotomy 1 to 2 weeks after re-staging.

### 2.2. Surgical Procedure

Single-lung ventilation was obtained by using a double-lumen tube. In cases of carinal or trachea-bronchial angle involvement, some technical intraoperative maneuvers were used: during the tracheo-bronchial resection and reconstruction, the tube was pulled back into the trachea, and a sterile endotracheal tube of small diameter was positioned under visual control by the surgeon in the contralateral main bronchus. Then the endotracheal tube was pulled down in the left main bronchus beyond the anastomosis after the reimplantation of the medial portion of the left main bronchus. and the tracheo-bronchial suture was thereby completed.

A lateral right “muscle-sparing” thoracotomy was preferred. In cases of chest wall involvement requiring resection and reconstruction, the type of thoracotomy was decided accordingly. The chest wall reconstruction was performed with polypropylene mesh/methylmethacrylate prosthesis.

When entering the pleural cavity after a previous lung resection, it was unavoidable to find pleural adhesions, which were released with great care in order to avoid visceral pleural tears and the contamination of the resulting pleural space. Sometimes, an extrapleural dissection was needed. Following this step, hilar vessels were immediately centrally controlled, usually intrapericardially. Attention was paid to keep as short as possible the bronchial stump and to reinforce it by vital pedicled mediastinal tissue (usually, pericardial fat pad). The chest wall was closed in airtight fashion, and the pleural space drained with a balanced system. Patients were managed in an intensive care unit overnight and moved to a stepdown unit when clinically appropriate.

All hospital survivors with pathological N2 disease received postoperative radiation therapy. Radiation therapy was given 4 to 6 weeks after the operation. The prescription dose was 50.4 Gy, administered in single, daily 1.8 Gy fractions on weekdays.

Survival and tumor recurrence were evaluated by patient follow-up. In particular, survival was defined as the interval between the day of CP and the time of the evaluation of this report, death, or loss to follow-up. Disease-free survival was calculated from the first day of surgical intervention until any event such as recurrence or metastases.

### 2.3. Statistical Methods

The two groups were compared with respect to the demographics and clinical outcomes using Student’s *t* tests or Fisher’s exact tests as appropriate. The survival probabilities were calculated via the Kaplan–Meier method and compared by using the long-rank test. Differences were considered significant when *p* < 0.05.

## 3. Results

**Figure 1:**
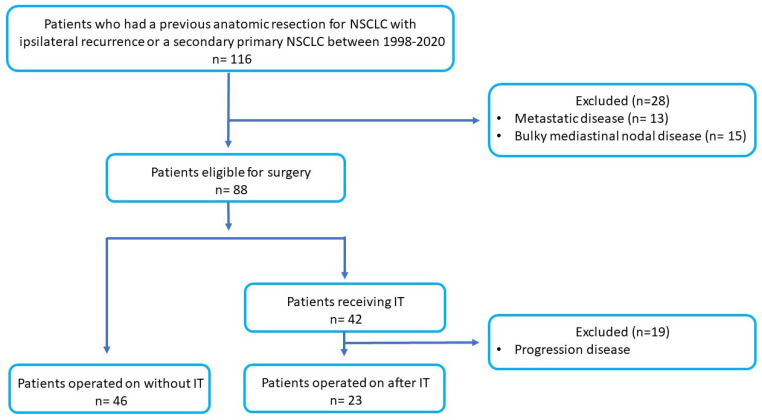
Flow diagram of population with NSCLC selected for this study. IT = induction therapy.

During the study period, a total of 116 patients who had a prior anatomic lung resection (segmentectomy, lobectomy, or bilobectomy) for NSCLC and presenting an ipsilateral recurrence or a second primary NSCLC were evaluated for possible CP. The flow diagram of selected population is reported in Figure 1.

Of these, 28 (24.1%) were turned away because of the presence of metastatic disease (n = 13) or bulky mediastinal nodal disease at the initial staging (n = 15). Of the remaining 88 patients, 46 were operated on without IT, and 42 (36.2%) received IT. Of this latter group, only 23 patients (19.8%) were operated on because 19 patients had progression of disease after IT (6 local and 13 distant). Thus, of the 116 observed patients, a total of 69 patients (59.5%) underwent CP (7.5% of all the performed pneumonectomies at our division): 23 patients (19.8%) underwent CP after IT (IT group), and this group was compared with the no-IT group (n = 46) which was comprised of patients who underwent CP without IT. Of the 69 patients, 22 (31.9%) had an ipsilateral recurrent tumor, and 47 (68.1%) had an ipsilateral second primary tumor.

In the IT group, 7 patients received chemoradiotherapy, 15 received chemotherapy, and 1 patient received radiation therapy. The mean number of cycles of chemotherapy received in the IT group was 4 (range, 3–9). The preoperative delay following IT before surgery was 4 weeks (range, 4–6 weeks). All patients receiving IT (n = 42), except 3 (due to severe hematological toxicity), had the planned induction protocol; no patient died during the study.

The baseline characteristics of the two groups are shown in Table 1.

The IT and no-IT groups had similar ages (*p* = 0.75) and gender distributions (*p* = 0.45) and similar mean preoperative forced expiratory volume in 1 min (*p* = 0.65) as well as diffusing capacity of the lungs for carbon monoxide (*p* = 0.69). Both histology and pathological stage were similar in both the groups (*p* = 0.34 and *p* = 0.38, respectively). The median time from the first operation to the CP was 25.5 months (range, 5–184 months). The first operation included 21 right upper lobectomies (3 sleeve lobectomies), 8 middle lobectomies, 13 right lower lobectomies, 5 bilobectomies, 12 left upper lobectomies, 5 left lower lobectomies, and 5 segmentectomies.

An associated resection of adjacent organs/structures was performed in 25 patients (36.2%) (Table 1). A chest wall resection and reconstruction were performed in 10 cases (14.5%), while a resection and reconstruction of the pulmonary artery were performed in 8 cases (11.6%), and a left atrium resection and a diaphragm resection were performed in 3 cases (4.3%). No differences were noted between the two groups (*p* = 0.12). A tracheal sleeve CP was performed in five cases (7.2%), two of which after IT (*p* = 0.23).

During surgical resection, some difficulties related to chemotherapy-induced fibrosis were encountered in the majority of IT-group patients; fortunately, these difficulties did not prevent the performance of the CP, and in fact, a complete resection was achieved in 100% of the entire population.

Intraoperative mortality was nil. Table 2 reports the incidence of postoperative morbidity and mortality.

Overall 30-day mortality was 7.2% (5 of 69). There was 8.7% (2 of 23) 30-day mortality in the IT group (one case of pneumonia after bronchopleural fistula and one case of acute respiratory distress syndrome-ARDS) and 6.5% (3/46) in no-IT group (one case ARDS after bronchopleural fistula; one case of cardiac arrest, and one case of acute respiratory failure with pneumonia) (*p* = 0.12).

No difference in terms of morbidity was registered between the two groups (*p* = 0.54), both for major (*p* = 0.12) or minor complications (*p* = 0.43). Overall morbidity was 37.7% (26 of 69), with 39.1% (9 of 23) in the IT group and 36.9% (17 of 46) in the no-IT group (*p* = 0.54); details are reported in Table 2.

In the IT group, bronchopleural fistulas occurred on the 29th postoperative day after a tracheal sleeve CP and were due to a dehiscence of the lateral portion of the anastomosis. A re-anastomosis was performed and reinforced externally by a pedunculated omental flap. The patient died on the 24th subsequent postoperative day from contralateral pneumonia. Additionally, a left diaphragmatic hernia occurred on the second postoperative day after a CP associated with a large diaphragmatic resection and reconstruction with a bovine pericardial patch. It was due to the disruption of two stitches. The hernia was repaired by using a new patch and assuring the absence of high tension in the diaphragmatic prosthesis.

In the no-IT group, one patient developed a bronchopleural fistula on the 15th postoperative day from a right CP. He was re-operated on and the small fistula was repaired by stiches and a pedunculated omental flap. The patient developed contralateral ARDS and died on the 32nd subsequent postoperative day. Additionally, a cardiac herniation occurred 24 h after a right tracheal sleeve CP and was due to the rupture of the pericardial prosthesis used to close a large pericardial defect. The prosthesis was fixed to the pericardium again by multiple separated stiches. The patient was discharged on the fifth postoperative day without complications. Finally, one patient had a transient ischemic attack on the sixth postoperative day which resolved itself after appropriate therapy.

In the IT group, 14 of 23 patients (52.1%) with clinical N2 disease were downstaged, with 2 patients achieving a complete response, and 10 patients who were downstaged from N2 to N1 disease. In the no-IT group, 19 of 46 patients (41.3%) were upstaged: 7 patients with N1 and 2 patients with N0 preoperative disease went to pathological N2, and 10 clinical N0 passed to pN1. Fifteen patients remained pN1, and two remained pN0. All 14 patients with pathological N2 disease (5 in the IT group and 9 in the no-IT group) received adjuvant radiation therapy.

**Figure 2:**
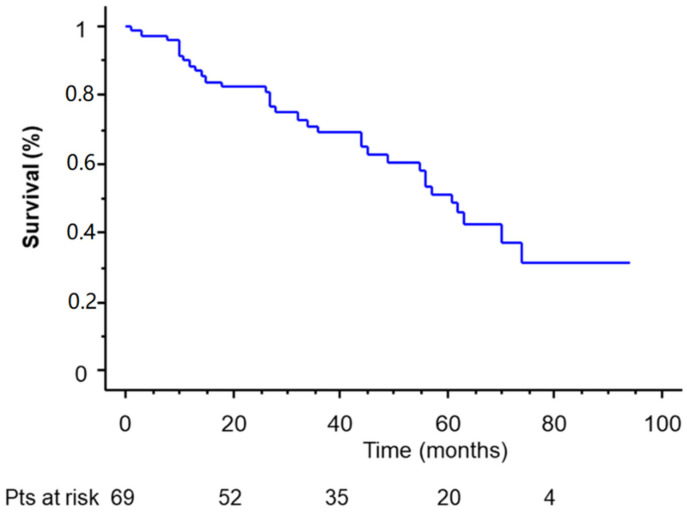
Overall survival of the entire population who underwent completion pneumonectomy.

Follow-up was completed for all patients. The mean overall survival was 35 months (range, 1–96 months; 95% confidence interval, 31.5–72.4). The overall 5-year survival for the entire population was 51.7% (Figure 2), while the overall 5-year disease-free survival was 36.2% (mean, 23 months; range, 3–94 months; 95% confidence interval, 18.2–49.7).

**Figure 3:**
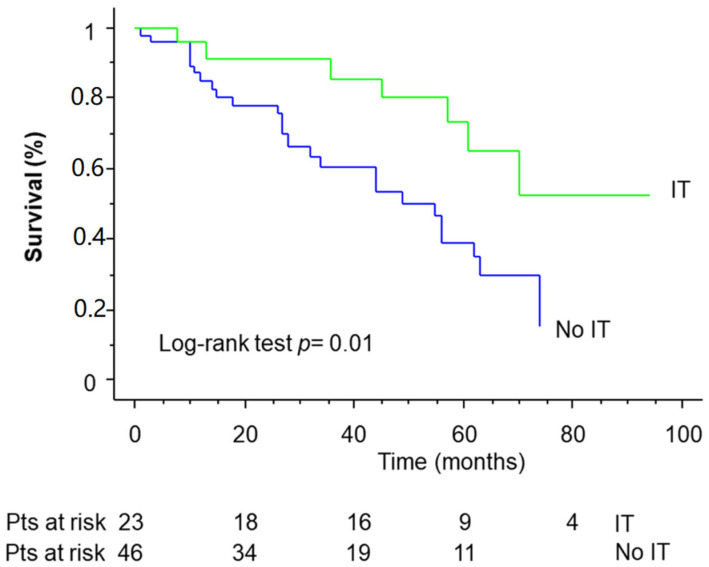
Kaplan–Meier survival of patients undergoing completion pneumonectomy, stratified by induction treatment.

The overall 5-year survival for the IT group was 69.8% compared with 39.2% for the no-IT group (*p* = 0.01) (Figure 3).

Factors influencing survival were IT (*p* = 0.01), extension of resection (*p* = 0.04), histology (*p* = 0.01), pathological stage (*p* = 0.03), and T and N factors (*p* = 0.2, both). Factors affecting survival at multivariate analysis included IT (*p* = 0.02) and histology (*p* = 0.03).

## 4. Discussion

CP remains a highly morbid operation with a significant risk of perioperative mortality. In our cohort of patients, 26 of 69 (37.7%) had postoperative complications and 5 (7.7%) died perioperatively, and these results are rather similar to prior published series [1,3,4,6,8,12,13,14,15]. In our series, major complications occurred in 5 patients (7.2%), while minor complications occurred in 21 patients (30.4%), and there was no difference between the two groups of patients (IT versus no-IT), which was also reported by Cardillo in a multicenter international study [3].

BPF occurred in two (2.9%) patients, both on the right side, one of which occurred with preoperative radiation. This rate is slightly lower than the majority of previous reports (2.7–13.3%) [3,5,7,12,16,17]. The following factors are considered the key factors impacting the outcomes of patients undergoing CP: surgical technique, the development of BPF, and the resulting morbidity and mortality [5,15]. Unfortunately, despite this knowledge, bronchial stump reinforcement with the coverage of a pedicled flap is not universally employed [5,15,18,19,20]. For our series, we employed a relatively uniform technique: for covering all bronchial stumps, which were kept as short as possible, we utilized regional mediastinal pedicled tissue (thymus or pericardial fat pad). Nevertheless, two patients developed BPF (one in each group), but there was no statistical difference between them.

Despite notable improvements in the management of the patients operated upon (anesthetic techniques, perioperative critical care, and attention to respiratory therapy) over the last decades, our results likely reflect the issue of a major operation in a frail patient population. For these reasons, a careful diagnosis and the preparation and patient selection are all crucial in order to reduce the morbidity rate. Preoperative staging, including whole body CT scan and PET scan, is high recommended as well as preoperative pathological diagnosis of the pulmonary lesion. Invasive mediastinal staging of suspicious nodes on CT/PET scan is also required to verify the correct mediastinal nodal staging and to identify those patients who must be definitively excluded from a surgical approach or who must receive IT. We suggest performing the mediastinal staging by EBUS and not by mediastinoscopy in order to avoid dense adhesions from primary surgery. The cardio-pulmonary functional evaluation is mandatory before CP: it has been demonstrated that a primary resection ensures better tolerance by the cardiopulmonary system of the second procedure compared with a one-stage pneumonectomy [7]. Moreover, a multidisciplinary tumor board discussion is paramount in order to ensure that surgical resection is the most appropriate treatment for every patient considered for CP for cancer. Preoperative optimization of patients undergoing CP is very important and includes smoking cessation, ensuring optimal pulmonary rehabilitation, and appropriate preoperative anti-infective therapy for patients with suspicion of associated pulmonary infections. This selective and planned approach demonstrated a slight improvement in outcomes compare with those in previous series [1,3,4,6,8,12,13,14,15].

Technical tips may be very important to reduce the risk of intra- and postoperative complications. Maneuvers such as intrapericardial blood vessel ligation, division of the bronchus first, bronchial reinforcement, and local application of glues and hemostatic agents may be very useful to avoid perioperative troubles.

Little has been published regarding the impact of preoperative treatments on CP, regardless of histology or indication, and also its influence on early and long-term outcomes. In fact, some authors have considered the administration of preoperative chemotherapy detrimental for postoperative outcomes [21,22], even in patients over 70 years old [23], but some large trials have shown no detrimental effect on morbidity and mortality for neoadjuvant chemotherapy [24,25].

Moreover, treatment of lung cancer can cause reduced quality of life, especially due to related respiratory or mediastinal complications such as swallowing or voice disorders. These complications are due to surgical injury or to exposition of cancer invasion of the recurrent laryngeal nerve and left vagus nerve. The impaired laryngeal mobility resulting from these nerve injuries may be dramatic after lung resection and nodal dissection and may be responsible for an important swallowing disorder, an ineffective cough, and the risk of pulmonary infections [26,27]. Glottic competence, and thus the resolution of these postoperative complications, may be quickly restored by some laryngoplastic techniques [28,29] with good outcomes in terms of quality of life for these patients.

It is well known that IT may increase the density of fibrosis and adhesions and this is particularly evident after a previous pulmonary resection. In our experience, we encountered increased difficulties in the surgical procedure in the IT group, but there was no difference between the two groups (IT versus no-IT). In terms of long-term results, in the study by White, 50% of patients received chemotherapy or chemoradiotherapy prior to CP [16]. With the constraints of small sample size, this does not appear to confer a survival advantage. On the contrary, in our experience, only 33.3% of patients undergoing CP received IT (15 cases of chemotherapy, 7 chemoradiation, and 1 radiation therapy). Although IT did not influence morbidity and mortality, it significantly influenced long-term outcomes. With an overall survival rate of 51.7%, patients who received IT had the best prognosis (69.8%) compared with those who did not receive IT (39.2%) (*p* = 0.01). The high survival rate, compared with the rates of 23% to 44.5% reported previously, was probably due to the inclusion of patients with early-stage lung cancer. Nevertheless, these favorable long-term results justify the procedure for the treatment of disease with severe prognosis.

The role of surgery after concurrent chemoradiation (CRT) versus RT alone for the treatment of locally advanced NSCLC has been evaluated [30,31,32,33] with largely uncertain results. In a retrospective study of the Dana–Farber/Brigham and Women’s Cancer Center, the authors showed that patients who were able to undergo surgical resection after CRT had an excellent outcome, and surgery remains a therapeutic option for properly selected patients [34].

Contrary to what is reported by other authors [3,6,8], in our series, patients with squamous cell carcinoma had worse survival (*p* = 0.03), but it was not influenced by IT. Although some authors find differences in terms of survival between patients who had recurrence versus those who had a second primary lung cancer [10,13,35], in our study, like in those reported by other authors, there was no difference in survival based on the interval between operations (*p* = 0.283) and also no difference between the two groups according to IT.

This study has several limitations: (a) although data were obtained from our single-institution, prospectively maintained database, the study remains retrospective in nature, and therefore, subject to bias in regard to data collection; (b) a selection bias could be considered our inability to capture patients with recurrent disease who were not offered or refused surgery for recurrent/second primary disease; (c) although this study was particularly focused on surgical outcomes (morbidity, mortality, and long-term outcomes) for ipsilateral recurrence/second primary NSCLC, an adequate adjustment to control the degree of bias, ideally using an SBRT control group, may be preferable. However, due to the rare entity of the event of NSCLC, a proper comparison may be difficult in a single-center experience; (d) moreover, we have employed a conservative and selective approach to the preoperative treatment groups (with and without induction treatment), which limited our sample size. Although propensity score matching might be a potential remedy to control the potential bias between the two treatment groups, it may risk overestimation due to the limited number of the study population [36].

## 5. Conclusions

Despite a selective approach, CP remains a demanding surgical procedure, associated with an acceptable morbidity and mortality, which should be performed in highly selected patients and in experienced thoracic surgery centers. In our experience, IT did not influence postoperative complications, while the absence of IT, advanced stages, and squamous cell carcinoma adversely influenced long-term outcomes. We think that correct patient selection is crucial before proposing CP as it is necessary to evaluate possible contraindications and to adopt technical details to reduce the complication rate.

## Figures and Tables

**Table 1:** Baseline characteristics of the study groups (n = 69).

Variable	IT (n = 23)	No-IT (n = 46)	*p* Value
Age (y ± SD)	62.70 ± 6.22	61.62 ± 5.86	0.75
Sex (male/female)	17M/6F	33M/13F	0.45
Side (right/left)	15/8	32/14	0.52
FEV1 (mean % predicted ± SD) FVC (mean % predicted ± SD)	81.72 ± 12.16 79.73 ± 16.88	80.48 ± 13.85 80.62 ± 14.67	0.65 0.73
DLCO (mean % ± SD)	76.30 ± 15.23	78.45 ± 16.08	0.69
Histology (%)			
Squamous	8 (34.8)	15 (32.6)	
Adenocarcinoma	13 (56.5)	25 (54.3)	
Others	2 (8.7)	6 (13.1)	0.34
Pathological stage (%)			
I	7 (30.4)	12 (26.1)	
II	11 (47.8)	25 (54.3)	
III	5 (21.7)	9 (19.5)	0.38
Extended resections			
Chest wall	3 (13.0)	7 (15.2)	
Pulmonary artery	3 (13.0)	5 (10.8)	
Left atrium	2 (8.7)	1 (2.2)	
Diaphragm	1 (4.3)	2 (4.3)	
Aorta Tracheal sleeve pneumonectomy	1 (4.3) 2 (8.7)	0 3 (6.5)	0.12 0.23

IT = induction therapy; SD = standard deviation; FEV1 = forced expiratory volume in 1 min; FVC = expiratory volume; DLCO = diffusing capacity of the lungs for carbon monoxide.

**Table 2:** Postoperative results.

Variable	IT (n = 23)	No-IT (n = 46)	*p* Value
Intraoperative mortality (%) 30-day mortality (%)	0 2 (8.7)	0 3 (6.5)	0.12
Morbidity (%)	9 (39.1)	17 (36.9)	0.54
Major	2 (8.7)	3 (6.5)	0.12
Diaphragmatic hernia	1 (4.3)	0	
Cardiac hernia	0	1 (2.2)	
Bronchopleural fistula TIA	1 (4.3) 0	1 (2.2) 1 (2.2)	
Minor	7 (30.4)	14 (30.4)	0.43
Pulmonary	4 (17.4)	8 (17.4)	
Cardiac	2 (8.7)	5 (10.8)	
Neurological ICU stay (days, median) Hospital stay (days, median)	1 (4.3) 1 8	1 (2.2) 1 7	1.00 0.83

IT = induction therapy; TIA = transient ischemic attack; ICU = intensive care unit.

## Data Availability

Available upon request.
